# Therapeutic Hints

**Published:** 1885-03

**Authors:** C. Hering


### THERAPEUTIC HINTS.

If well chosen remedies do not act, give—

PSORINUM, when the patient shows a psoric taint.

OPIUM, when of a torpid nature.

CARBO VEG., when he is weak, emaciated, with feeble pulse.

LAUROCERASUS, when he is nervously agitated.

All women who are prone to abort should take Sepia or Zincum.

